# Curriculum Innovations: Implementing a Neuropalliative Care Curriculum for Neurology Residents

**DOI:** 10.1212/NE9.0000000000200021

**Published:** 2022-11-29

**Authors:** Sonya Taryn Gleicher, Caroline Jeanette Hurd, P. Annie Weisner, Ali Marisa Mendelson, Claire J. Creutzfeldt, Breana L. Taylor

**Affiliations:** From the Department of Neurology (S.T.G., P.A.W., C.J.C., B.L.T.), Division of Geriatrics and Gerontology (C.J.H.), Department of Medicine, University of Washington; and Hospice and Palliative Care (A.M.M.), Kaiser Permanente Washington, Seattle.

## Abstract

**Background and Objectives:**

Neuropalliative care is an emerging interprofessional field that aims to improve communication and quality of life for all people affected by serious neurologic disease. Teaching neuropalliative care skills is a key objective for neurology residencies, and the Accreditation Council for Graduate Medical Education requires proficiency in palliative care. The objective of this study was to describe a novel longitudinal multimodal curriculum in neuropalliative care communication and evaluate its feasibility and outcomes.

**Methods and Curriculum Description:**

We designed a multimodal curriculum focused on neuropalliative care communication skills using as our theoretical foundation transformative learning theory. We implemented this program for neurology residents at a single academic institution over the course of their 3-year training. Residents underwent (1) 3 communication workshops using VitalTalk modules and simulated patient encounters, (2) 3 or more observed clinical encounters with structured faculty feedback, and (3) at least 3 annual neuropalliative care lectures. We evaluated the effect on learners' self-assessed confidence in neuropalliative care skills with preworkshop and postworkshop questionnaires.

**Results and Assessment Data:**

In 2021, 14 of 20 eligible residents attended our workshops and completed the preworkshop questionnaire, and 12 of those completed the postworkshop questionnaire. After the workshop, a larger proportion of residents (75%, 9/12) agreed or strongly agreed that they felt confident leading family meetings compared with before the workshop (57%, 8/14). While more than 90% of residents felt confident recognizing patient and family emotions both before and after the workshop, the workshop improved their confidence in responding to such emotions. Still, some residents neither agreed nor disagreed (42%, 5/12) about feeling confident in responding to emotions after the workshop, and many commented on wanting more training in this area.

**Discussion and Lessons Learned:**

The successful implementation and high attendance among eligible participants demonstrate the feasibility of our longitudinal multimodal neuropalliative care curriculum. The evaluation of intervention outcomes suggests that residents' confidence in neuropalliative communication skills improved. Our study shows that VitalTalk is a tool that can be adapted to teach neuropalliative communication skills for neurology residents, and this program can be easily adopted by other neurology training programs.

Neurologic disorders represent the leading cause of disability-adjusted life-years worldwide and account for approximately 1 in 5 global deaths.^1,2^ The high prevalence of distressing symptoms associated with neurologic disease including an often uncertain prognosis requires physicians to be skilled in a spectrum of care beyond patients' biomedical needs.^3^ Neuropalliative care is an interprofessional field that focuses on maximizing quality of life of patients and their families by addressing their physical, psychosocial, spiritual, and cultural concerns.^4^ Data suggest that neuropalliative care skills such as serious illness communication may improve patient satisfaction and medical outcomes,^5^ and the Accreditation Council for Graduate Medical Education (ACGME) requires proficiency in palliative care.^6^

Unfortunately, our current models of training do not adequately prepare neurologists to meet patients' palliative care needs, and most neurology program directors rate residents' neuropalliative care skills and training as poor.^6,7^ Reasons for this gap between recommendations and practice may include residents' busy schedules, residents seeing communication as an intrinsic skill rather than one that can be taught or learned, and difficulty identifying faculty to provide formal feedback on palliative care or communication skills.^7^

Despite facing similar challenges, other training programs such as medical oncology have integrated palliative care training by using validated experiential curricula designed to improve communication skills, such as the online platform VitalTalk.^8^ Neuropalliative care and training trail behind, although research suggests that palliative care needs are as prevalent or even more prevalent in neurologic illness compared with other serious illnesses.^3^ For example, symptom burden in advanced Parkinson disease is similar to that of metastatic cancer, and caregivers of people with neurologic disease have similar, if not higher, rates of distress and burnout as caregivers of patients with cancer.^3^ Reasons for palliative care consultation are different for patients hospitalized with neurologic disease compared with those hospitalized with cancer, suggesting unique needs for neuro-specific palliative care.^9^

Based on these needs, a curriculum at the University of Washington (UW) was formed for neurology residents. The foundational framework of this curriculum was informed by VitalTalk and transformative learning theory. VitalTalk is a nonprofit organization which develops evidence-based trainings to help clinicians communicate effectively with patients living with serious illness.^8^ Transformative learning is a primarily adult theory of learning which asserts that learning is the process of making meaning of one's experiences. It emphasizes taking learners out of their comfort zone so that they can question their old ways of thinking, transform their mindset, and develop new habits of mind, all of which lead to changes in behavior.^10^ This theoretical foundation combined with VitalTalk led to our curriculum's multimodal and highly experiential nature.

## Problem Statement

Given the prevalence of palliative care needs and unique considerations of neuropalliative care, strategies specific to neurology are needed to promote neuropalliative skills. There is a gap in our current understanding of how to optimally teach and assess skills in neuropalliative care longitudinally for neurologists and trainees. In this study, we examine a multimodal curriculum that uses evidence-based trainings from VitalTalk to help clinicians communicate with patients living with serious illness. We apply a transformative learning framework to understand whether this curriculum helps residents challenge their old schemes of communication and generate new practices in neuropalliative care. We aim to determine whether this approach is feasible and effective for training neurology residents in neuropalliative care.

## Objectives

We developed and piloted a longitudinal multimodal curriculum in neuropalliative care (MCNC) at UW using evidence-based educational materials including VitalTalk. The goal of this curriculum was to provide residents with the communication skills they need to meet the needs of persons affected by serious neurologic disease. In this study, we disseminate the details of this curriculum and evaluate its feasibility and learning outcomes so that other educators may adopt similar training programs.

In collaboration with VitalTalk and UW faculty, we identified palliative care skills that any practitioner of neurology should master and established the following educational objectives:

1. To coach residents in developing actionable skills and tools for family meetings, including sharing serious news and discussing goals of care.
2. To teach residents how to recognize and respond to patient and family emotions.
3. To establish a common language for communicating with patients, families, and clinicians around palliative care needs.
4. To improve residents' confidence and comfort in providing neuropalliative care.

## Methods and Curriculum Description

### Curriculum Participants

The curriculum was implemented with all UW neurology residents over the course of their 3-year neurology training from postgraduation year (PGY) 2 to PGY4. This included 8 adult neurology and 2–3 pediatric neurology residents per class. Workshop attendance was required for all eligible residents (not postcall, sick, or on vacation).

### Curriculum Structure

The backbone of the curriculum comprised annual summer workshops and observed patient encounters. For PGY3 and PGY4 residents, who attend didactics together, we implemented an alternating curriculum such that 1 of 2 summer workshops was offered each year. At the beginning of their PGY3, residents were assigned a track based on which summer workshop was being offered that year. Figure depicts the general curriculum design.

**Figure:**
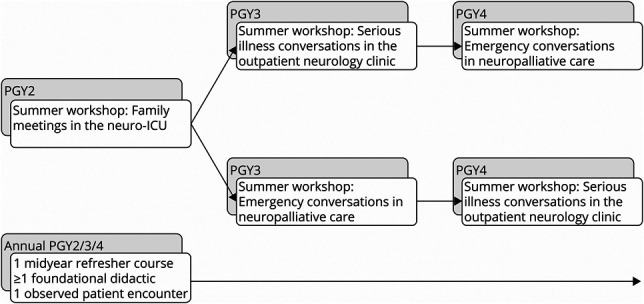
Longitudinal Workshop Schedule for Neuropalliative Care Curriculum With Alternating Schedule for PGY3–4 Residents ICU = intensive care unit; PGY = postgraduation year.

Additional foundational neuropalliative care education provided included the following:

1. At least 1 annual foundational neuropalliative didactic on amyotrophic lateral sclerosis, neuro-oncology, the neuro-intensive care unit (ICU), and general neuropalliative care.
2. One additional didactic each for multiple sclerosis and neurodegenerative diseases is planned.
3. Each of the 2-hour summer workshops was followed by 1-hour midyear refreshers, which served as opportunities to check in with learners on how they were applying their neuropalliative care skills.
4. As the curriculum evolved, we observed how emotionally taxing providing neuropalliative care can be for residents. Residents were encouraged to explore the challenging conversations and feelings that arise when caring for sick or dying patients at monthly palliative care discussion groups (called “death rounds”).^11,12^ We also set up quarterly peer support check-ins for residents in this curriculum.

### Curriculum Implementation

We conducted summer workshops in May 2017, June 2018, August 2019, August 2020, and September 2021, along with midyear refreshers in the winter of each year. All workshops were scheduled during residents' required didactic time. There were 4–7 learners and 1 facilitator per group. Workshops were facilitated by UW internal medicine or neurology faculty with palliative care postgraduate or fellowship training. All faculty members were provided with a faculty workshop manual. We recruited simulated patients (SPs) from the UW School of Medicine, who had been previously trained with VitalTalk, and reviewed a character guide before the encounter. Although workshops were initially conducted in person, we transitioned to video conference during the coronavirus disease 2019 (COVID-19) pandemic for social distancing purposes.

### Curriculum Content

Curriculum content was informed by transformative learning theory and validated teaching materials from VitalTalk,^13^ which we applied specifically to neuropalliative scenarios in the outpatient clinic, emergency department, and neuro-ICU (Figure). Using transformative learning, we felt it was important not only to teach learners VitalTalk toolsets through didactics but also to supplement this with experiential learning in the form of observed simulated and real patient encounters with feedback.

Summer workshops covered key elements from our learning objectives. Table 1 summarizes the content covered in each of the summer workshops.

**Table 1:** Summary of Content From Neuropalliative Care Curriculum Workshops^8^

Summer session no.	Content focus	Teaching materials	SP encounter
1. PGY2	Family meetings in the neuro-ICU	7-step family conference framework	Discuss preferences for life-sustaining treatment with daughter/son of an 84-y-old man with advanced dementia admitted to ICU for progressive respiratory failure
		NURSE framework	
2. PGY3/PGY4	Serious illness conversations in the neurology outpatient clinic	PAUSE talking map	Discuss early goals of care with a 60-y-old man with glioblastoma who is hesitant to make decisions about intubation or resuscitation
3. PGY3/PGY4	Emergency conversations in neuropalliative care	GUIDE framework	Share serious news and discuss late goals of care with a spouse of a 45-y-old formerly healthy woman who meets criteria for brain death after intraparenchymal hemorrhage
		REMAP framework	

Abbreviations: ICU = intensive care unit; PGY = postgraduation year; SP = simulated patient.

Each summer workshop contained 4 main elements:

#### Preworkshop Self-Learning

Learners were emailed before the workshop to complete assigned online materials, including the VitalTalk Tips smart phone application, and a simulated family meeting.

#### Group Content Review

At the workshop, facilitators reviewed neuropalliative care skills and frameworks with learners in small groups. Table 2 presents the summer workshop structure, which was adapted from VitalTalk.

**Table 2:** Components of Summer Workshops From Neuropalliative Care Curriculum^8,23^

Activity	Goals	Steps
Introductions/ice breaker	Set tone for the session Get to know one another	1. Facilitator reviews agenda and provides context for the session 2. Facilitator builds enthusiasm by bringing the curriculum close to the point of care 3. Learners share names and personal details outside medicine
Content review	Reinforce skills from self-directed modules	1. Facilitator explains content for session 2. Facilitator emphasizes how content could be adapted to patient/family needs
Small group ground rules/being a good observer	Create safe space Ease tensions around role play Set expectations	1. Learners share past experiences with role play 2. Learners discuss ground rules, including confidentiality, constructive feedback, respect when others speak, and time-outs 3. Facilitator explains the structure of simulated practice 4. Facilitator explains how to be a good observer
Simulated practice	Adapt content to clinical scenarios Practice communication skills	1. Learners each have ∼15 min to practice with simulated patients or family members
Take home points	Answer remaining questions Reinforce skills	1. Facilitator asks group reflection questions 2. Learners ask any remaining questions 3. Learners write one thing they learned during that session and how they will incorporate it into their next neuropalliative encounter. Learners then share this take home point with the group
Wrap-up	Remind learners that practice continues during real clinical encounters	1. Facilitator reviews the VitalTalk Tips smart phone application 2. Facilitator explains opportunities to practice neuropalliative skills, such as family meetings and medical updates 3. Facilitator emphasizes observed encounters and feedback forms

#### Simulation

Learners observed one another's conversations during SP encounters. Facilitators would time out at key learning opportunities to allow learners to ask questions, receive real-time feedback, and rewind the conversation to try a new skill. Since peer feedback is an important part of this learning process, we provided residents concrete tools to improve their observation and feedback skills.^14^

#### Observed Patient Encounters

Residents conducted annual observed patient encounters in clinical settings and received structured feedback from faculty. Initially, observed patient encounters were not reported by all residents, so we made this a graduation requirement and designed a faculty feedback form (eTable 1, links.lww.com/NXG/A562), which residents submitted as evidence of completion. To ensure faculty engagement and reliable feedback for observed patient encounters, we reviewed the goals of this curriculum with participating faculty and provided a “cheat sheet” to familiarize the faculty with the VitalTalk modules that residents were learning in the workshops.

### Curriculum Assessment

Our main outcomes were feasibility of the program and resident learning/knowledge (Kirkpatrick level 2)^15^ using preworkshop questionnaires conducted at the August and September 2021 workshops and identical postworkshop questionnaires (eTable 2, links.lww.com/NXG/A562) conducted approximately 3 months later. Questionnaires were administered through the online survey platform SurveyMonkey (Momentive Inc., San Mateo, CA).^16^

The assessment measured key objectives for our curriculum. Table 3 summarizes how the questionnaire items correlated with learning objectives. Questionnaires consisted of 9 items, including 7 statements which assessed learners' self-assessed competence and confidence in neuropalliative care on a Likert scale from 1 (“strongly disagree”) to 5 (“strongly agree”), 1 open-ended item, and 1 item to collect data on how many family meetings residents had led. This outcome assessment corresponds to the Level 2: learning on the Kirkpatrick model training evaluation system.^15^

**Table 3:** Correlation Between Learning Objectives and Questionnaire Items From the Assessment of the Neuropalliative Communication Skills Curriculum

Learning objective	Associated questionnaire item
To coach residents in developing actionable skills and tools for family meetings, including sharing serious news and discussing goals of care	Q1. I feel confident leading a family meeting independently for a patient with a serious neurologic illness Q4. I feel confident giving serious news
To teach residents how to recognize and respond to patient and family emotions	Q2. I feel confident recognizing patient and family emotions during a family meeting Q3. I know how to respond to patient and family emotions during a family meeting
To establish a common language for communicating with patients, families, and clinicians around palliative care needs	Q5. I feel confident eliciting a patient's values Q6. I feel confident discussing end-of-life care
To improve residents' confidence and comfort in providing neuropalliative care	All questionnaire items

### Data Availability

Anonymized data not published within this article will be made available by request from any qualified investigator.

## Results and Assessment Data

### Learner Characteristics

Overall, 14 neurology residents (7 PGY2 and 7 PGY3–4, all others were postcall, sick, or on vacation) participated in the 2021 summer workshops. Before the workshop, 8 residents reported having led less than 5 family meetings independently, 4 had led 5–10, and 2 had led more than 10.

### Questionnaire Response Rate

Of the 14 residents who attended workshops, 7 PGY2 residents submitted preworkshop questionnaires and 5 submitted postworkshop questionnaires. All 7 of the PGY3–4 residents submitted preworkshop and postworkshop questionnaires. All questionnaires were completed in full.

### Questionnaire Outcomes

Learner responses to the preworkshop and postworkshop questionnaires are summarized in Table 4. In the postworkshop questionnaire, a larger proportion agreed or strongly agreed to feeling confident about certain communication skills compared with the preworkshop questionnaire. Residents' confidence recognizing patient and family emotions did not change substantially after the workshop, and their confidence giving serious news decreased. In the free text option, residents identified the following as areas they wanted to work on in the future: eliciting values, neuroprognostication, responding to emotions, and end-of-life care.

**Table 4:** Preworkshop and Postworkshop Questionnaire Responses From the Neuropalliative Care Curriculum

Questionnaire statements	Preworkshop responses agree or strongly agree (N = 14), n (%)	Postworkshop responses agree or strongly agree (N = 12), n (%)
Q1. I feel confident leading a family meeting independently for a patient with a serious neurologic illness	8 (57.1)	9 (75.0)
Q2. I feel confident recognizing patient and family emotions during a family meeting	13 (92.9)	11 (91.7)
Q3. I know how to respond to patient and family emotions during a family meeting	6 (42.9)	7 (58.3)
Q4. I feel confident giving serious news	9 (64.3)	7 (58.3)
Q5. I feel confident eliciting a patient's values	7 (50.0)	8 (66.7)
Q6. I feel confident discussing end-of-life care	7 (50.0)	8 (66.7)

### Additional Feedback on Curriculum

Several learners provided unsolicited feedback on the curriculum. They were grateful to have practiced what can be uncomfortable conversations in a simulated environment. One learner recalled, “As uncomfortable as it was in the moment, I am so, unbelievably glad we had our simulation…I ended up leading a pretty unexpected transition to comfort care conversation…I kept faltering, and ultimately ended up relying so heavily on the phrases we were trying the other day.” Having these skills allowed learners to appreciate a sense of accomplishment in neuropalliative care: “This is the first time I have had a family thank me in this setting…and the first time I felt I had a direct role in helping them get to a place where it seemed to be a ‘good death.’”

## Discussion and Lessons Learned

This longitudinal MCNC meets the ACGME milestone requiring neurology residents to acquire proficiency in palliative care and develop skills in patient-centered and family-centered communication.^6^ Using evidence-based teaching methods and content, its experiential MCNC design is supported by research in both medical education and adult learning, which suggests that the most successful curricula allow people to be active agents in their own learning.^7,10,17^ Our program incorporates recommended teaching methodologies including SP encounters, direct observation and coaching, acronyms, and pocket cards accessible through VitalTalk.^7,17^

The theoretical background for our curriculum's teaching methods and structure is informed by transformative learning. We found that there were several components to optimally apply this theory to neuropalliative education: (1) experiential learning such as SP encounters and observed patient encounters provided learners the opportunity to critically examine how they discuss serious news and synthesize new perspectives in communication; (2) toolsets and learning modules from VitalTalk and the feedback from peers and mentors guided residents toward taking on intended communication skills from these patient encounters; and (3) we supported learners while we took them out of their comfort zone because challenging residents in these ways can be detrimental to learning without an environment of safety and respect.^18^ For this reason, key parts of the curriculum included training faculty and residents on providing constructive feedback and support groups where residents could process these intense experiences. We are encouraged in our application of transformative learning theory by learner feedback. One resident experienced a substantial shift in the perspective of end-of-life conversations and wrote an unsolicited email about their “first time” being involved in a “good death” experience.

We evaluated curricular outcomes by assessing changes in residents' learning (level 2 on the Kirkpatrick model training evaluation system).^15^ Data collected from preworkshop and postworkshop questionnaires suggest that the program increased residents' confidence in practicing certain neuropalliative skills. It is possible that skills in which residents reported unchanged or diminished confidence may reflect the Dunning-Kruger effect,^19^ whereby individuals become more aware of their knowledge deficiencies as they gain expertise.

Our training program demonstrates other outcomes related to educational interventions such as feasibility and adoption. Feasibility, an implementation outcome which measures the extent to which an innovation can be made,^20^ is demonstrated by our successful recruitment and retention of learners and implementation of the curriculum over the past 4 years. We encouraged workshop attendance by incorporating the program into protected learning time, minimized resources required by using an alternating curriculum, and promoted faculty investment by disseminating background on the purpose of our curriculum. Although ensuring that residents practice their communication skills in real clinical settings was challenging at first, we resolved this by tracking observed patient encounters with a faculty feedback form and making this a requirement for graduation. Finally, successful adoption of the program, defined as learners' intention to use this practice,^20^ is supported by residents' feedback on how helpful the training had been in their clinical encounters.

This study has several limitations. Although feasibility is demonstrated by the successful implementation of our curriculum, analysis of its efficacy is limited by the small sample size. Some factors potentially contributing to the sample size include our relatively small residency class, difficulty attaining 100% attendance because of the high clinical demands on residents and competing work hours, and data limited to the most recent workshops. In addition, the number of resident responses was different for our presurveys and postsurveys, limiting the accuracy of comparison between the 2 groups. We could not match individual residents between the samples because they were anonymous. Our questionnaires assess changes in learners' attitudes toward neuropalliative care but do not measure higher Kirkpatrick levels such as changes in clinical behavior or patient outcomes.^15^ This workshop was developed and conducted at a single institution and, therefore, may not be automatically generalizable to other institutions. For example, some medical centers may not have neurologists trained in palliative care, but one potential solution would be to partner with outside facilitators, now that virtual learning has become more universal. We believe that most neurology residencies can adapt this format to suit their individual needs and resources.

This experience has several implications for education in neuropalliative care. We effectively adapted VitalTalk to teach neuropalliative communication skills and believe this can be feasibly performed at other institutions. Most VitalTalk training programs for physicians comprised a single or limited number of sessions, but we believe that the 3-year longitudinal nature of our curriculum promotes long-term changes in behavior through deliberate practice and reinforcement over time.^21,22^ We, therefore, encourage other institutions to consider adopting longitudinal neuropalliative curricula. Based on this experience and learner feedback, we believe that experiential learning, which pushes learners out of their comfort zone in a supportive environment, puts them in the best position to change their practice and recommend educators to consider transformative learning as a theoretical foundation for their curricula.

Future directions for our program may include improving our sample size by expanding the training program to include our neurology fellows and faculty and a multicenter study including neurology programs outside UW. We are also continuously improving our SP scenarios, including incorporation of diverse patient backgrounds, common socioeconomic barriers, and current challenges such as the recent COVID-19 pandemic. Finally, we collaborated with multiple faculty members in the development of this curriculum and believe that its content can be further improved by involving even more stakeholders in its design, including patients and families, neurology subspecialists, residents, and interprofessional team members.

In conclusion, we present a longitudinal MCNC, which provides simulated content, validated teaching resources, and recommended pathways to deliver that content. We hope our description of this curriculum can help other residency programs apply similar interventions.

## Appendix Authors

**Table:**

Name	Location	Contribution
Sonya Taryn Gleicher, MD, EdM	Department of Neurology, University of Washington, Seattle	Drafting/revision of the manuscript for content, including medical writing for content; analysis or interpretation of data
Caroline Jeanette Hurd, MD	Department of Medicine, Division of Geriatrics and Gerontology, University of Washington, Seattle	Drafting/revision of the manuscript for content, including medical writing for content; major role in the acquisition of data; study concept or design; analysis or interpretation of data
P. Annie Weisner, MD, PhD	Department of Neurology, University of Washington, Seattle	Drafting/revision of the manuscript for content, including medical writing for content; study concept or design
Ali Marisa Mendelson, MD	Hospice and Palliative Care, Kaiser Permanente Washington, Seattle	Drafting/revision of the manuscript for content, including medical writing for content; study concept or design
Claire J. Creutzfeldt, MD	Department of Neurology, University of Washington, Seattle	Drafting/revision of the manuscript for content, including medical writing for content; major role in the acquisition of data; study concept or design; analysis or interpretation of data
Breana L. Taylor, MD	Department of Neurology, University of Washington, Seattle	Drafting/revision of the manuscript for content, including medical writing for content; major role in the acquisition of data; study concept or design; analysis or interpretation of data

## Glossary

ACGME: Accreditation Council for Graduate Medical Education
COVID-19: coronavirus disease 2019
ICU: intensive care unit
MCNC: multimodal curriculum in neuropalliative care
PGY: postgraduation year
SP: simulated patient
UW: University of Washington
